# Student’s Self-Reported Experience of Soundscape: The Link between Noise, Psychological and Physical Well-Being

**DOI:** 10.3390/ijerph21010084

**Published:** 2024-01-11

**Authors:** Florence Renaud, Ingrid Verduyckt, Tiffany Chang, Adriana Lacerda, Cecilia Borges, Annelies Bockstael, Rachel E. Bouserhal

**Affiliations:** 1 Department of Psychology, Faculty of Arts and Science, University of Montreal, Montreal, QC H3C 3J7, Canada; florence.renaud@umontreal.ca; 2 School of Speech-Language Pathology and Audiology, Faculty of Medicine, University of Montreal, Montreal, QC H3N 1X7, Canada; tiffany.chang@umontreal.ca (T.C.); adriana.lacerda@umontreal.ca (A.L.); 3 Department of Psychopedagogy and Andragogy, Faculty of Arts and Science, University of Montreal, Montreal, QC H2V 2S9, Canada; cecilia.borges@umontreal.ca; 4 Department of Health and Care, Artevelde University of Applied Sciences, 9000 Ghent, Belgium; annelies.bockstael@arteveldehs.be; 5 Electrical Engineering Department, École de Technologie Supérieure, Montreal, QC H3C 0J8, Canada; rachel.bouserhal@etsmtl.ca

**Keywords:** noise, classrooms, children, bodily reactions

## Abstract

Extensive research has shown that noise has detrimental effects on learning in classrooms, yet schools remain noisy environments. In addition, little is known about the students’ insight into their subjective reaction to noise. Students’ awareness of noise, as well as their perception of its effects on their affective and bodily states, remain unanswered. In the current study, the self-reported experience of noise and reaction towards noise, which was collected by way of a questionnaire, was assessed for 408 students in primary and secondary schools in Québec. Results suggest that about half of the students experience affective and bodily reactions to noise, and students who report having a negative affective reaction to noise are also more prone to report feeling this noise in their bodies. The results of this study offer a comprehensive picture of the students’ subjective (affective and bodily) state in relation to noise in schools.

## 1. Introduction

The quality of a school’s environment is important to assure optimal learning and students’ overall well-being. However, research has shown school environments can be suboptimal; when students and teachers are asked about the quality of their school environment, noise is frequently reported as a major problem [1,2,3]. Worldwide, noise levels in schools are alarming, with average levels above the 35–50 dB recommendation for optimal learning environments [4,5,6]. In the last few decades, extensive research on the effects of noise on children has been conducted. Findings have shown that noise in schools is a problematic issue because it can affect the students’ physical health [7,8], their academic performance [7,9], and their well-being [7,10].

A recent scoping review of the effects of classroom acoustic conditions on primary school children’s mental well-being suggests noise in classrooms can have detrimental effects on children’s well-being, as established by children’s emotions, behavioral symptoms, and quality of life [10]. More recently, a systematic review of the effects of ventilation-related sounds on students showed the negative effects of fan noise and human-made sounds during natural ventilation on student performance [11]. Although these findings are of concern, research in this area remains limited, with a focus on primary school-age children and on sound sources labeled as “negative” or coming from the outside environment [12]. However, studies have shown that noise coming from inside the schools and generated by the human activities in the school are the most frequent noises students are exposed to [13]. Students are sensible judges of their acoustic environment; they are aware of the noise, and they can be annoyed by it [2,14,15,16,17,18]. Haines and Stansfeld [19] reported that the students’ noise annoyance was strongly associated with the students’ perception of the noise interfering with the task they were undertaking. Annoyance was related to the fact that noise made it hard to think and work. Other studies showed a similar link between noise annoyance and perceived disturbance or interference with academic tasks [14,18]. Several studies have also underlined that subjective factors interplay with the effect of noise on individuals [20]; for example, students’ perception of the pleasantness of their learning environments’ sound environment is not linked to objective measurements of sound levels [13,21]. Furthermore, Boman and Enmarker [22] found that noise annoyance was related to noise influencing the students’ emotions and their body sensations. The students report that noise makes them irritated, tired, or stressed and that they become tense and have headaches. Similarly, other authors [17,23,24] found that noise is perceived as emotionally and physically painful by children. This set of studies suggests noise has an impact on the students’ affective state and that the students are conscious of these effects. Interestingly, Astolfi and colleagues [25] showed that students’ affective state has an impact on their perception of noise. In this study, they assessed classroom acoustics, students’ overall well-being, and students’ subjective perception of noise. Their results showed differences in the subjective reaction towards noise of generally happy and unhappy students. In classrooms with bad acoustics (reverberation times of 0.9–1.4 s), happy students showed a greater tendency to judge the noise as a disturbance than unhappy students or happy students in classrooms with good acoustics (reverberation times of 0.5–0.8 s). Conversely, unhappy students do not report being disturbed by noise, but when they are in classrooms with bad acoustics, they have lower scores of well-being, self-esteem, emotional health, and enjoyment of school than happy students or unhappy students in classrooms with good acoustics [25,26]. Similarly, Klatte and colleagues [27] found that classrooms with bad acoustics negatively influenced students’ judgment of their relation to their peers and teachers. These findings suggest students might not be fully aware of the effects of noise on well-being. The present study seeks to contribute to the understanding of the interrelations between noise and well-being by documenting subjective reactions—emotional, physical, and behavioral—as perceived by the children who inhabit the classroom’s soundscape.

Auditory processing and emotional regulation are closely connected, ref. [28] proposed in a theoretical article that the intimate connection between the auditory cortex and emotional cortical and subcortical regions of the brain is the result of human evolution. Indeed, according to these authors, the subjective interpretation of a soundscape promotes the survival of the species through the preference and avoidance of different environments. Emotions and noise are also related to body sensations [23,29]. Noise can make one’s ears or head hurt. It can also be physically uncomfortable, presumably analogous to an emotional reaction. It is well documented that children have bodily and affective reactions to noise [17,22,23,24]. Previous research focused mainly on physical health (e.g., cortisol levels, blood pressure) or well-being (e.g., quality of life, mental health) separately, without investigating the possible parallel between physical and emotional aspects of noise reaction. Understanding a student’s subjective reaction to noise by exploring the potential links between emotional, physical, and symptomatologic factors is essential to adapting noise management strategies in schools to promote well-being. Addressing well-being in relation to noise is important not only in itself but also because well-being is known to be associated with learning, motivation, and academic achievement [30,31,32].

In the context of academic performance, research has shown that noise impairs performance at school by reducing the intelligibility of speech of the teacher [33,34,35], by distracting the students, and by impairing their cognitive performance [7,9]. Importantly, cognitive impairment by noise is not directly associated with the appraisal of the noise by students [36,37,38]. According to appraisal theory, appraisal is a process in which the individual’s values, evaluations, goals, control, and agency about a situation cause an affective reaction [39,40]. Hygge [37] showed that children’s ratings on mood and irritation scales about noise did not reflect the effect of noise on memory tasks. Similarly, Massonnié and colleagues [36] showed that noise annoyance and noise interference were two different constructs. Annoyance is a negative affective reaction towards noise, such as displeasure, anger, or stress [7]. Noise interference, on the other hand, is a mechanism where noise taxes cognitive resources, disrupting a current task or chain of thoughts [36]. Thus, noise interference has more to do with cognitive impairment. Although noise annoyance and noise interference are correlated to some extent, their distinction might help better understand inter-individual differences in students’ reactions to noise [36]. It can explain why some students may only suffer cognitively from noise, exhibiting a decrease in academic performance without feeling annoyance towards the noise. On the other hand, other students may suffer only affectively from noise; performing well cognitively but with impaired well-being. In the worst-case scenario, some students are affectively and cognitively impaired by noise. This last case could imply (1) that perceived noise interference can affect annoyance, (2) that annoyance can affect interference, or (3) that noise annoyance and noise interference are two parallel phenomena occurring simultaneously in some people. Some studies suggest students are aware noise interferes with their work and are thus annoyed by it [14,18,19], supporting statement (1). However, Stallen [38] showed that interference is not the only defining factor of noise annoyance. As presented in their theoretical framework, annoyance reactions towards noise have an affective component that is independent and goes beyond the fact that noise is perceived as interfering with a task or a goal. The current state of the literature does not allow the exclusion of any of these three possibilities. Understanding the cognitive, affective, and physical reactions of students to noise is thus really important to promote optimal learning and well-being in school environments.

Additionally, the coping strategies students utilize have an influence on their subjective reaction to the noise and interference that noise has with the ongoing activity. Indeed, a perceived noise disturbance is annoying to the extent that the individual does not perceive possibilities to act on mitigating the noise [38]. This puts children at greater risk of negative noise effects [41]. They have less control over their environment than adults, and they have immature coping strategies [42]. For instance, Dellve, Samuelsson, and Waye [23] found that students often reported not knowing how to cope with noise. Still, some students were able to report a few coping strategies, including leaving the noisy area, covering their ears, reporting the noise to a teacher, or using cognitive strategies such as concentrating harder or distracting themselves [23]. Similarly, other studies reported that some students use mind-wandering or daydreaming as a strategy to avoid the noise [22,36]. These coping strategies can explain a part of the difference between noise interference in the task and noise annoyance. For example, mind-wandering interferes with the ongoing task but eases the affective disturbance of noise [36]. Moreover, Boman and Enmarker [22] reported that noise annoyance could be attenuated by the students taking part in the noise. Similar to mind-wandering, this strategy interferes with academic tasks but relieves the students from the negative effects caused by noise. Overall, students have not been taught how to properly cope with noise and lack strategies to do so, yet they were still able to come up with strategies such as covering their ears and moving away from the noise [23,24,43,44].

The appraisal of a sound as a noise requires a subjective judgment that varies between individuals [38]; it follows that sounds do not have the same effect on every student. A better understanding of how sounds in schools are appraised by students on a cognitive, affective, and physical level is important. It could explain important inter-individual differences and support the development of individualized noise-coping strategies to guide efforts in creating optimal learning environments for a diverse set of students.

The present study aims to contribute to this understanding by investigating the subjective perception of sounds in schools using a self-reported questionnaire assessing students’ affective state, bodily state, and coping reactions to sounds. This study addresses an overlooked component of noise research: subjective perception. Thereby, the current study is exploratory, investigating (1) the number of students subjectively affected by noise, (2) the nature of subjective reaction, and (3) related behaviors and symptoms.

## 2. Methods

### 2.1. Participants

Participants in this study were students from 9 different Quebec schools. The classes were selected from a pool of teachers and students who participated in a hearing prevention activity organized by the School of Speech-Language Pathology and Audiology, University of Montreal. The participants were from primary schools (grades 4, 5 and 6) and secondary schools (grades 2 and 5). Participation in this study was optional and anonymous. The final sample population includes 408 students. The project was approved after an ethical review by the Ethical Committee for research in Education and Psychology of the University of Montreal.

### 2.2. Materials

A translated and adapted version of the Inventory of Noise and Children Health (INCH), developed by Waye and colleagues [45], was used. In this questionnaire, students are invited to answer questions related to their appraisal of three different types of sounds, namely: (1) other children being angry and yelling; (2) loud and intense sounds such as screaming and banging; (3) scraping and screeching sounds. For each sound category, students were asked to answer how often they heard them on a five-point scale ranging from “Almost never” to “Always”. They also had to indicate their bodily reaction to each sound category by circling the relevant body parts on a drawing of a child. Their affective reaction to each sound category was then assessed by two scales ranging from glad/safe–sad/afraid and from kind/friendly–angry/irritated. The range of reactions on each scale was represented by five drawings of a child with different body postures and facial expressions. Students were then invited to answer if they used four common noise coping strategies (go away, cover the ears, tell the teacher, and raise one’s own voice), and if so, how often on a five-point scale ranging from almost never to always. Covering one’s ears was not included as a strategy in the INCH addressed to secondary school students, as a small pilot study with secondary students for the adaptation of the INCH revealed this strategy was irrelevant for this age group. Lastly, they were asked if they had experienced the following three symptoms in the past few days: headache, tummy ache, or a hoarse voice, and answered on a five-point scale ranging from never to often. The questionnaire wording can be found in Table 1.

### 2.3. Procedure

Students were handed out the paper questionnaire by their teacher during a regular class where 10–15 min were allotted to answering the questions. Children were allowed to return a blank questionnaire.

### 2.4. Statistical Analysis

Data were analyzed using SPSS25 (IBM, Armonk, NY, USA). Descriptive statistics were used to describe the findings of the students’ self-reported questionnaire. To synthesize the students’ answers regarding where they felt noise in their body, the circled body parts were regrouped into three main areas: head, mid-body, and lower body. The area of the head includes sensations reported in the ears, forehead, eyes, eyebrows, mouth, and lower face. The mid-body area includes sensations reported in the chest, heart, shoulders, arms, wrists, hands, and stomach. Finally, the lower-body area includes sensations reported in the legs and feet. The three main sub-areas were coded for in a binary way: a score of 0 was attributed if no part of the body was marked, and a score of 1 was given if one or more body parts belonging to the sub-area were marked. Chi-squared tests ( $\chi^{2}$ ) were run to explore associations between affective reaction, bodily reaction, coping strategies, physical symptoms, gender, and school grade/levels. When the expected frequencies in cells were too low for the chi-square test, Fisher’s exact tests were used. If the Fisher test failed, the Monte Carlo method was used to simulate Fisher’s exact test results. Significance was set at an α of 0.05 and was adjusted for some analysis using the Bonferroni method for multiple comparisons, which is explicitly indicated when applicable. A Spearman’s rank correlation was performed to assess the relationship between the glad/safe–sad/afraid and the kind/friendly–angry/irritated scales.

## 3. Results

Out of 409 questionnaires returned, only one was completely unanswered and excluded. Missing or uninterpretable data were found in 207 questionnaires (50.6%). The reasons for missing data were on one part, the absence of three questions in 17 questionnaires (4%), due to printing issues, omitted questions by the student, or the presence of more than one answer from the student to a question (e.g., circling in several alternatives on the five-point scales). On average, each of the questions had 4.7% missing or uninterpretable data, and students had, on average, one missing or uninterpretable answer in their questionnaires. All questionnaires, even if not complete, except for the entirely blank one, were taken into account in the analyses (*n* = 408). Missing or uninterpretable data explains why the total n differs for some of the analysis results. Detailed sample characteristics are presented in Table 2.

Girls and boys are evenly distributed overall and between grade levels. Overall, 46.1% of the participants reported being girls, 44.1% being boys, while 5.9% chose the “other” alternative. Due to the low percentages of “other”, further analyses regarding gender will be limited to comparing girls versus boys. The majority of the samples (n = 279; 68.4%) come from primary school students.

### 3.1. Level of Occurrence of Three Types of Sounds

**Figure 1:**
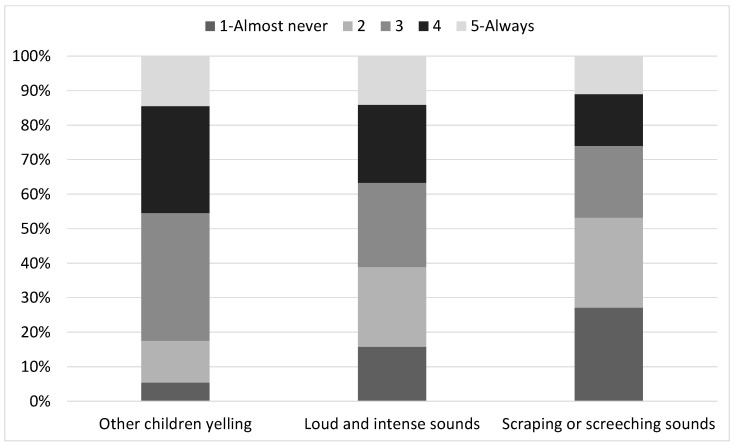
The perceived level of occurrence of three types of sounds as reported by students on a 5-point Likert scale (1 = Almost never; 5 = Always).

Figure 1 summarizes the proportion of students reporting each level of occurrence for the three different types of sounds.

As can be seen, “Other children yelling” is the type of sound that most children report hearing frequently, with 45.5% of students scoring 5 (always) or 4, while only 17.5% report hearing this type of sound at a low frequency (e.g., level 1 or 2). The second most frequently heard type of sound is “Loud and intense sounds”, of which 36.6% of children report hearing at a frequency of 4 or 5. However, slightly more children (38.9%) report only hearing these sounds at a frequency of 1 or 2. Furthermore, 26% of children report hearing “Scraping and screeching sounds” at a frequency of 4 or 5, while a majority (63.2%) report hearing them at a frequency of 1 or 2. Chi-squared tests show that there is a significant association between students’ school level (primary versus secondary) and the perceived frequency of hearing “Other children yelling” ( $\chi^{2}\left(4,N=387\right)=63.38,p<0.001$ ) and “Loud and intense sounds” ( $\chi^{2}\left(4,N=385\right)=25.69,p<0.001$ ) and “Scraping and screeching sounds” ( $\chi^{2}\left(4,N=399\right)=12.65,p=0.011$ ). While students in primary school report hearing “Other children yelling” and “Loud and intense sounds” more frequently than secondary school students, secondary school students report hearing “Scraping and screeching sounds” more frequently than primary school students. Chi-squared tests were run to explore if there were any differences between girls and boys, but no statistical difference was found, indicating that boys and girls perceive the level of occurrence of the different sounds in a similar way.

### 3.2. Affective Reactions to Three Types of Sounds

**Figure 2:**
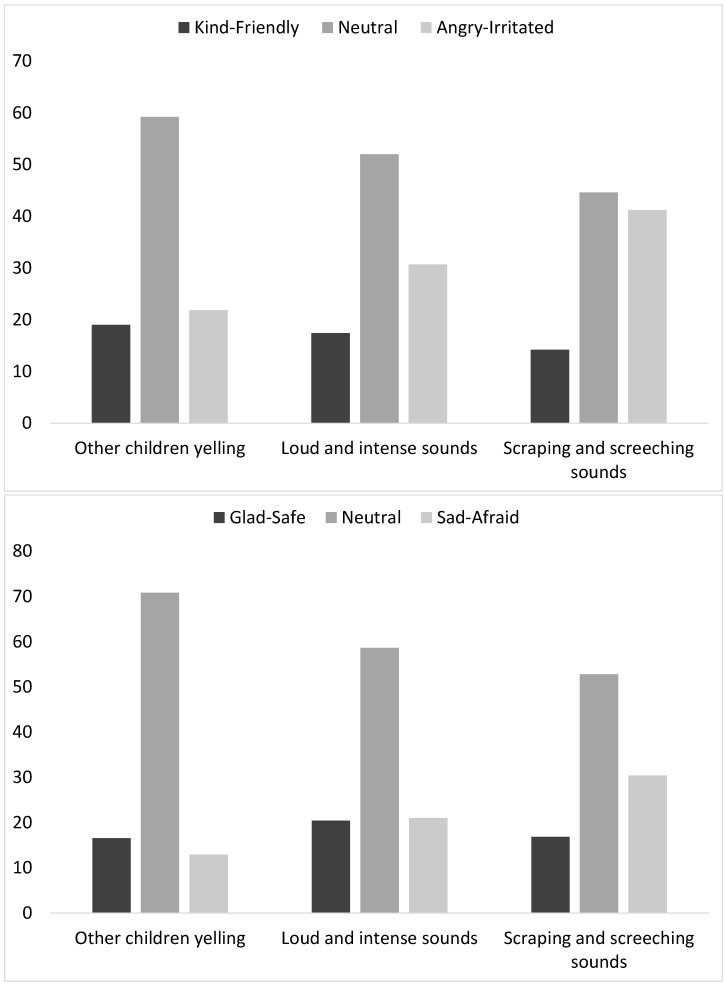
Affective reactions to three types of sounds.

Frequencies of students’ responses regarding their affective reactions to the different types of sounds are reported in Figure 2.

A Spearman’s rank correlation was performed to assess the relationship between the glad/safe–sad/afraid and the kind/friendly–angry/irritated scales for the three types of sounds. The two scales show a significant but moderate correlation for each type of sound ( r(372)=0.39,p<0.001 for “Other children yelling”, r(361)=0.54 , p<0.001 for “Loud and intense sounds”, and r(381)=0.47,p<0.001 for “Scraping and screeching sounds”, indicating that the two scales measure related but not similar constructs. Regarding both the glad/safe to sad/afraid and the kind/friendly to angry/irritated scales, the majority of students report a neutral level of reaction (3) to the three types of sounds, mostly so for “Other children yelling” (70.8% on the glad/safe–sad/afraid scale and 59.2% on the kind/friendly–angry/irritated scale), while the positive (1;2) and negative (4;5) reactions on both scales are quite evenly distributed. Regarding “Loud and intense sounds”, the distribution of positive and negative reactions is quite even on the glad/safe to sad/afraid scale, but on the kind/friendly to angry/irritated scale, there is a higher proportion of children reporting negative than positive reactions (30.7% negative vs. 17.4% positive). Finally, “Scraping and screeching sounds” yields the highest proportion of negative reactions on both scales (30.4% negative on the glad/safe–sad/afraid scale and 41.2% negative on the kind/friendly–angry/irritated scale). Chi-squared tests show no significant differences between affective responses in primary and secondary school students. However, there is a significant difference between boys and girls for “Other children yelling” on the glad/safe to sad/afraid scale (Fisher’s exact: p=0.043 ), and on both scales for “Loud and intense sounds” (Fisher’s exact: p=0.001 on the glad/safe to sad/afraid scale, and $\chi^{2}\left(4\right)=10.646$ , p=0.031 on the kind/friendly to angry/irritated scale). More girls react negatively to both of these types of sounds, while more boys react positively. “Scraping and screeching sounds” do not yield significantly different reactions in boys and girls.

### 3.3. Bodily Reactions to the Three Types of Sounds

As explained in the analysis section, students’ reports of bodily sensations in reaction to sounds were grouped into three main body areas for the statistical analyses: the head, the mid-body, and the lower body. All three sound types taken together, the majority of reported sensations are in the head area (28.9–48%), while the least sensations are reported in the lower body area (1.7–7.6%). Overall, 38.6% of the students (n = 157) report feeling sounds such as “Other children yelling” in their body, 60% (n = 242) report feeling “Loud and intense sounds” in their body, and 66.7% (n = 257) report feeling “Scraping and screeching sounds” in their body. Detailed data are presented in Table 3.

**Figure 3:**
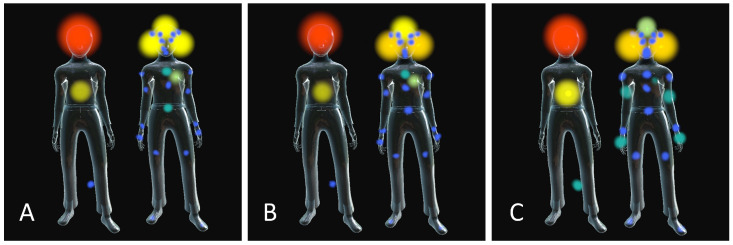
Heat-maps of bodily reactions to the three types of sounds. Mapping of bodily reactions to (**A**) “Other children yelling”; (**B**) “Loud and intense sounds”; and (**C**) “Scraping and screeching sounds”. In each picture, the body map to the left shows children’s answers as synthesized into three main areas: head, mid-body, and lower body. The body map to the right shows children’s detailed answers representing all body parts children circled in.

Chi-squared tests show that the bodily reactions in the mid-body and lower body vary as a function of the type of sound ( $\chi^{2}\left(2,N=649\right)=10.273,p=0.006$ and $\chi^{2}\left(2,N=648\right)=16.105$ , p<0.001 ). Post hoc tests with a Bonferroni adjusted *p*-value of 0.006 show that the difference is significant for “Loud and intense sounds” versus “Scraping and screeching sounds” and for “Other children yelling” versus “Scraping and screeching sounds” for both mid- and lower-body areas ( p<0.001 ) with a more prominent feeling in the mid-body and lower body for “Scraping and screeching sounds” as compared to the other sounds. Students’ grouped and distinct answers regarding bodily reactions to the three types of sound are illustrated in Figure 3 in the form of a body heat-map, where the color and size of the colored dots indicate the proportion of answers given for each type of sound.

The body maps on the right, picturing the distinct answers to each type of sound, reveal fine differences between types of sounds that the synthesized data fails to demonstrate. For example, the head area reactions to “Other children yelling” are evenly distributed through the head/brain area and the ears, while the ear area is more often reported in relation to the head/brain area for “Loud and intense sounds”, and even more so for “Scraping and screeching sounds”. For “Scraping and screeching sounds”, the mid-body area is regrouping reactions felt in the arms and hand in a greater proportion than for the two other types of sounds. Moreover, specifically for “Other children yelling” and “Loud and intense sounds”, some children had circled the heart area with some specifically drawing a heart shape.

The proportion of students reporting bodily reactions to sounds also differs as a function of gender and school level (primary vs. secondary) (Table 3. Chi-squared tests show that significantly more girls than boys report a bodily reaction to each type of sound. Indeed, 46.3% of the girls vs. 32.2% of the boys report a bodily reaction to “Other children yelling” ( $\chi^{2}\left(1,N=368\right)=7.607,p=0.006$ ). For “Loud and intense sounds, 65.4% of the girls vs. 54.8% of the boys report a bodily reaction ( $\chi^{2}\left(1,N=365\right)=4.297$ , p=0.038 ), and for “Scraping and screeching sounds”, 71.4% of the girls vs. 60.1% of the boys ( $\chi^{2}\left(1,N=363\right)=5.095,p=0.024$ ) report a bodily reaction.

More primary school students than secondary school students report a bodily reaction to “Other children yelling” (41.4% vs. 32.6%; NS), to “Loud and intense sounds”, (65.5% vs. 46.9%; $\chi^{2}\left(1,N=404\right)=12.52,p<0.001$ ), and to “Scraping and screeching sounds” (71.2% vs. 56.7%; $\chi^{2}\left(1,N=387\right)=8.00,p=0.006$ ).

### 3.4. Association between Affective Reactions and Bodily Reactions

**Figure 4:**
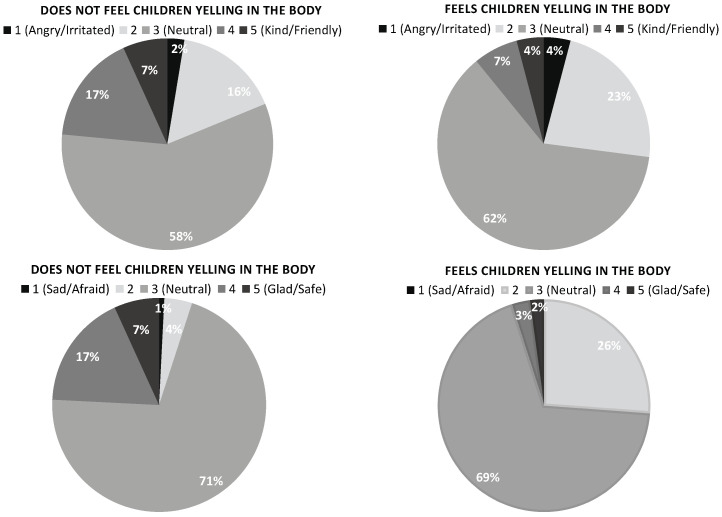
The proportion of children reporting different levels of affective reactions to children yelling as a function of having or not bodily reactions to these sounds.

There is a clear association between bodily reactions and affective reactions on the glad/safe to sad/afraid scale for the three types of sounds: “Other children yelling” ( $\chi^{2}\left(4\right)=55.918,p<0.001$ ), “Loud and Intense sounds” ( $\chi^{2}\left(4\right)=39.366,p<0.001$ ), and “Scraping and screeching sounds” ( $\chi^{2}\left(4\right)=52.147,p<0.001$ ). The affective reactions measured by the kind/friendly to angry/irritated scale are also significantly associated with the presence of bodily reactions for the three types of sounds: “Other children yelling” ( $\chi^{2}\left(4\right)=11.287,p=0.024$ ), “Loud and intense sounds” ( $\chi^{2}\left(4\right)=18.842,p<0.001$ ), and “Scraping and screeching sounds” ( $\chi^{2}\left(4\right)=63.399,p<0.001$ ). Post hoc tests with a Bonferroni correction show that children who report bodily reactions to the sounds also tend to report negative affective reactions to these sounds. Detailed results for “Other children yelling” are presented in Figure 4. Detailed results for “Loud and intense sounds” and “Scraping and screeching sounds” can be found in the Supplementary Material.

### 3.5. Noise Coping Strategies

**Figure 5:**
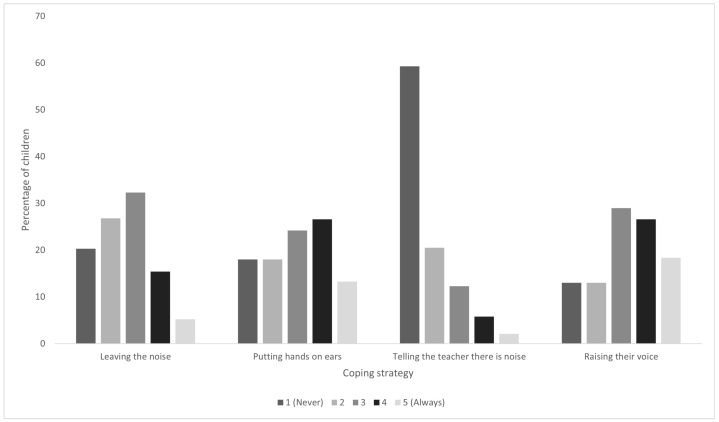
Students’ answers regarding the use of coping strategies on the 5-point frequency scale.

Responses of students’ frequency of use of different coping strategies towards noise on the five-point scale ranging from “never” (1) to “always” (5) are presented in Figure 5. The most used strategies are “Raising one’s voice” (45% scoring either of the two highest levels of the five-point scale) and “Putting hands on ears” (39.9% scoring either of the two highest levels of the five-point scale), while the least used strategy is “Telling the teacher there is noise”, with 79.8% scoring either of the two lowest levels of the five-point scale. There is a difference in how boys and girls use the coping strategies “leaving the noise” ( $\chi^{2}\left(4\right)=20.47,p<0.001$ ), and “raising one’s own voice” ( $\chi^{2}\left(4\right)=10.77,p=0.029$ ). Post hoc tests reveal that boys more often than girls report a level 1 (never) on leaving the noise, but slightly more girls than boys report a level 2. Regarding “raising one’s own voice”, more boys than girls report levels 2 and 5 (always), while more girls report a level 4. In addition, students in secondary school report using the strategies leaving the noise ( $\chi^{2}\left(4\right)=31.56,p<0.001$ ) and telling the teacher (Fisher’s exact: p=0.002 ) less often than students in primary school.

### 3.6. Association between Coping Strategies and Bodily and Affective Reaction

Chi-square analyses show that the use of coping strategies is positively associated with bodily reactions to sounds. Students who report feeling noise in their bodies use some coping strategies more frequently than students who do not feel noise in their bodies. The strategy “putting the hands on the ears” (only explored in primary school students) was the only strategy to be associated with bodily reactions to all three types of sounds, respectively, “Other children yelling” ( $\chi^{2}\left(4\right)=19.06,p=0.001$ ), “Loud and intense sounds” ( $\chi^{2}\left(4\right)=21.19,p<0.001$ ), and “Scraping and screeching sounds” ( $\chi^{2}\left(4\right)=15.07$ , p=0.005 ). For “Other children yelling” and “Loud and intense sounds”, post hoc analyses show that significantly more students who do not report bodily reactions score at a level 1 or 2 ( p<0.001 ), while a significantly higher proportion of those reporting a bodily reaction to these sounds scores at a level 4 or 5 ( p<0.001 ). For “Scraping and screeching sounds”, a higher proportion of students reporting no bodily reaction to these sounds score at a level of 1 ( p<0.001 ), while a higher proportion of those reporting a bodily reaction to these sounds scores at level 4 or 5 ( p<0.001 ). The strategies “leaving the noise” and “telling the teacher” were associated with bodily reactions to “Other children yelling” ( $\chi^{2}\left(4\right)=14.67,p=0.005$ and Fisher’s exact: *p* = 0.008, respectively), and “Loud and intense sounds” ( $\chi^{2}\left(4\right)=23.85,p<0.001$ and Fisher’s exact: p=0.042 , respectively). Post hoc testing shows that a higher proportion of children with no bodily reaction to “Other children yelling” or “Loud and intense sounds” score at a level of 1 for leaving the noise ( p<0.001 ), while a higher proportion of those reporting a bodily reaction to these sounds scores at a level of 4 for this strategy ( p<0.001 ). For “Loud and intense sounds”, there is also a higher proportion of children with bodily reactions to these sounds that score 5 for this strategy ( p<0.003 ). Finally, the strategy “raising my voice” was only associated with bodily reactions to “Loud and intense sounds” ( $\chi^{2}\left(4\right)=18.41,p=0.001$ ), with more children reporting no bodily reactions to these sounds scoring at level 1 ( p<0.001 ) and 3 ( p=0.010 ) on “raising my voice”, while more children reporting bodily reactions score at level 2 and 5 ( p<0.001 ).

Spearman’s rank correlations show that the use of coping strategies is weakly but significantly associated with reporting higher frequencies of negative affective reactions toward sounds with correlation coefficient values ranging between 0.109 and 0.286 (see Table 4).

Leaving the noise was positively associated with higher levels of negative reactions on both affective scales for all types of noise ( p<0.005 ). Putting the hands on the ears was positively associated with higher levels of negative reactions on both affective scales for all types of noise ( p<0.005 ) except for the kind/friendly to angry/irritated continuum regarding “Other children yelling”. Telling the teacher was positively associated with higher levels of negative reactions on both affective scales for all types of noise ( p<0.05 ) except for the kind/friendly to angry/irritated continuum regarding “Scraping and screeching sounds”. Finally, increasing one’s own voice was only significantly and positively associated with higher levels of negative reactions on the kind/friendly to angry/irritated continuum regarding “Loud and intense sounds” ( p=0.05 ).

### 3.7. General Symptoms

**Figure 6:**
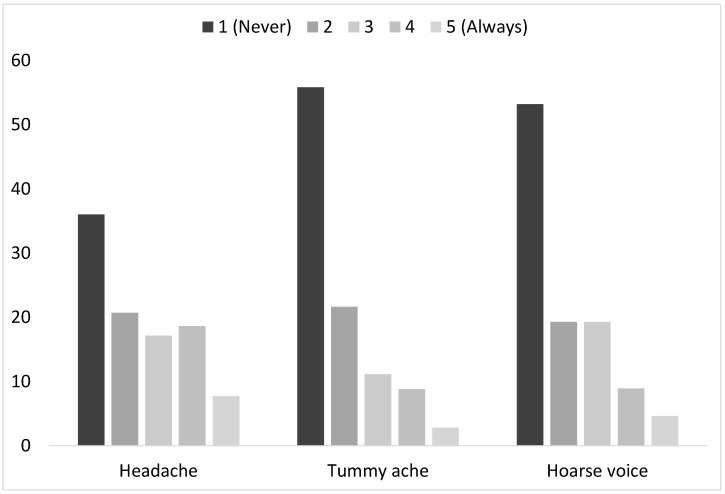
Student’s experience of symptoms at school as reported on the 5-point frequency scale.

The amount of respondents for each frequency level on the general symptom scales is presented in Figure 6. As seen in the figure, the highest proportions of answers are in the low frequencies, with a majority of the students scoring at the lower levels of the scale (1 and 2) for headache (56.7%), for tummy ache (77,4%), and for hoarse voice (72.5%). That said, the most frequent symptom that is experienced is headache, with 26.3% of children scoring at the higher end of the scale (4–5) for this symptom compared to 13.5% for hoarse voice and 11.6% for tummy ache. Chi-square analyses show that the general symptoms of headaches and tummy aches are associated with gender ( $\chi^{2}\left(4\right)=22.47,p<0.001$ and $\chi^{2}\left(4\right)=17.08,p=0.002$ , respectively) with a higher proportion of boys than girls (45% vs. 27.2%) reporting headache at a frequency level of 1 and a higher proportion of girls than boys (25.5% vs. 13%) expressing headache at a frequency level of 4. A higher proportion of boys than girls (64.4% vs. 48.1%) reported tummy ache frequency at a level of 1. See Supplementary Material for detailed results. There are no significant differences between primary and secondary school children regarding frequency and type of symptoms.

### 3.8. Association between General Symptoms and Bodily and Affective Reaction

Chi-square analyses show that the experience of general symptoms is associated with bodily reactions to sounds. Feeling “Other children yelling” in one’s body is associated with the following symptoms: headaches ( $\chi^{2}\left(4\right)=53.29,p<0.001$ ), tummy ache ( $\chi^{2}\left(4\right)=22.67,p<0.001$ ), and a hoarse voice ( $\chi^{2}\left(4\right)=14.31,p=0.006$ ). Post hoc analyses reveal that a larger proportion of students who report not feeling “Other children yelling” in their body report never experiencing headaches ( p<0.001 ), tummy aches ( p<0.001 ), and a hoarse voice ( p<0.001 ), while a larger proportion of students who do report feeling “Other children yelling” in their body scores a 4 on the frequency scale of experiencing headaches ( p<0.001 ).

Feeling “Loud and intense sounds” in one’s body is associated with the following symptoms: headaches ( $\chi^{2}\left(4\right)=22.85,p<0.001$ ) and tummy ache ( $\chi^{2}\left(4\right)=32.94$ , p<0.001 ). Again, post hoc analyses show that a larger proportion of students who report not feeling ”Loud and intense sounds” in their body report never (1) experiencing headaches ( p<0.001 ) and tummy aches ( p<0.001 ).

Feeling “Scraping and screeching sounds” in one’s body is also associated with the symptoms of headaches ( $\chi^{2}\left(4\right)=15.71,p=0.003$ ) and tummy ache ( $\chi^{2}\left(4\right)=15.71$ , p=0.003 ). Again, post hoc analyses show that a larger proportion of students who report not feeling “Scraping and screeching sounds” in their body report never (1) experiencing headaches ( p<0.001 ) and tummy aches ( p<0.001 ).

Spearman’s rank correlations show that general symptoms are weakly but significantly associated with reporting higher frequencies of negative affective reactions towards sounds with correlation coefficient values ranging between 0.108 and 0.212 (see Table 5 for detailed results).

The reported frequency of the symptom headache is positively associated with the frequency of negative affective reactions on the glad/safe–sad/afraid scale for all types of sounds ( p=0.005 ) and on the kind/friendly–angry/irritated scale for “Loud and intense sounds only” ( p=0.005 ). Tummy ache is positively associated with the frequency of negative affective reactions on both affective scales for all three sounds ( p=0.05 ), while a hoarse voice is positively associated only with “Scraping and screeching sounds” on the kind/friendly–angry/irritated scale ( p=0.05 ) and on “Loud and intense sounds” and “Scraping and screeching sounds” on the glad/safe–sad/afraid scale ( p=0.05 ).

## 4. Discussion

The present study aims to explore affective and bodily reactions as well as physical symptoms from classroom noise exposure as perceived by students. The study adds to previous research by investigating students’ subjective reactions to noise in school and by suggesting a comprehensive body sensation map from students’ answers. Students report being exposed to noise in their school environment. Ratings of the frequency with which noises are heard show that other children yelling is the most frequently heard noise, followed by loud and intense sounds and, finally, scraping and screeching sounds. It is unclear if these results are in agreement with past research because of the lack of consideration of internal noise sources in schools. Past studies mainly focused on external noise sources (traffic, airplane, construction, etc.), whereas this study investigated internal noise sources (yelling students, loud and intense sounds (door banging, footsteps in the corridor, falling objects, etc.), and scraping and screeching sounds (squeaking door, shuffling of classroom furniture, etc.)). In noise studies assessing internal noise, noise categories are not constituted the same way, limiting comparison. For example, Astolfi and colleagues [1] and Visentin and colleagues [13] used noise categories based on where the noise takes place (in the classroom, in the corridor, in the neighboring classrooms, and outside), and Connolly and colleagues [46] had more detailed noise sources, including noise from inside the classroom, noise from inside the school, and mechanical noise. That said, scarce past research tends to show similar internal noise patterns as this study, i.e., noise made by students’ voices (talking loudly, screaming) seems to be more frequent than mechanical noise or furniture noise [1,46]. Visentin and colleagues [13] also found that sounds generated by the children in the classroom (including both sounds such as voices and furniture scraping) were not only the most frequently heard by the children in their study, but these sounds were also deemed unpleasant more often than pleasant or neutral.

The students’ responses suggest a big part of them are negatively affected by noise. All noise types taken into account, 37% of the students report having a negative affective reaction to noise, and 55% of them feel the noise in their body. These results corroborate past findings on emotional and physical disturbance of noise in students [13,17,18,22,23,24,43,45,47]. Past research has shown body sensations to be a significant part of children’s reactions to noise [17,22,23,24]. However, to our knowledge, our study is the first study on noise perception to present a comprehensive body map of where students report feeling different types of noises, unsurprisingly with the head, especially the ears and the forehead being reported, but also, the heart, and stomach area, and, for Scraping and screeching sounds, the arms and legs. Understanding noise-related body sensations might be really important to promote optimal well-being and learning in schools. Furthermore, affective reactions and body sensations are associated. Students who report having a negative affective reaction to noise respond more frequently that “yes, I feel noise in my body”. That could be explained by the fact that students who have somatic complaints about noise are more likely to have a negative affective state regarding noise in school (e.g., if noise makes my ears hurt, I am irritated by the noise). Another explanation that cannot be ruled out is that the affective state caused by the noise comes with body sensations that are intrinsic to that affective state [29]. Unfortunately, the results of this study do not make it possible to draw conclusions on the nature and direction of the link between affective noise reaction and bodily noise reaction.

Some variation did appear between noise types. Negative affective reactions and body sensations were more frequent for scraping and screeching sounds and less frequent for other children yelling. Again, it is unclear how these results fit into the existing literature on noise. Past research has shown that of the noises coming from inside the school, the noise made by other students (shouting, yelling, talking, running) is more problematic and annoying than noise from ventilation or furniture (banging doors, shuffling tables and chairs) [3,16,22]. However, Bulunuz and colleagues [14] found no clear differences between noise made by students and furniture noise in regards to annoyance, and Connolly and colleagues [46] found the opposite, as mechanical noise and furniture noise were more annoying than the noise made by students inside the school. Other studies, such as [1,13], have not distinguished between type of noise according to the noise source, but rather as to whether the noise comes from inside the classroom, outside the classroom, or outside the school. In this study, other children yelling was less affectively and physically disturbing but was the most frequently heard noise, and scraping/screeching was the most affectively and physically disturbing as the less frequently occurring noise. This is consistent with previous findings suggesting that the frequency of occurrence of noise is not related to the degree of annoyance caused by the noise [13,15,46] and that intermittent noise is more annoying [1,13]. Subjective reactions to noise, such as annoyance, affective reaction, and bodily reaction, are influenced by its “subjective natures”: non-acoustical factors such as noise sensitivity, attitude, expectations, and perceived control influence these reactions [18,38]. Infrequent, unpredictable, and uncontrollable noise could thus be more disturbing. In addition, students who take part in the yelling may not report it or experience it as being emotionally and physically disturbing [22].

The coping strategies evaluated in this study were rarely used by the students. This could be explained by the type of coping strategies chosen to be evaluated in this study (it did not include cognitive strategies such as concentrating harder, daydreaming, and mind-wandering) or the fact that students do not know how to cope with noise. However, some students state that they always use coping strategies, and a student reporting using one coping strategy is more likely to report using other coping strategies (except raising the voice). The result of this study suggests primary school students more frequently use the strategies of leaving the noise, putting their hands on their ears, and telling the teacher. However, secondary school students report more often the use of the strategy of raising their voices. This could be explained by the fact raising one’s voice in a noisy environment is an involuntary and often unconscious behavior (e.g., the Lombard effect) [48]. Older students may be more aware of this behavior, and thus, they can report it in the questionnaire, whereas younger students, who may unconsciously exhibit that behavior, do not report it. That said, raising the voice was overall the most frequently used strategy, as reported by the students in this study. However, this strategy is problematic: (1) students raising their voices are contributing to the already high noise levels in their school environment [49], (2) raising a voice in the long-term can have detrimental effects on students’ and teachers’ vocal health [50]. Furthermore, 59.3% of students report never telling the teacher about the noise, making that strategy the least used one. This result is surprising as one might think students complain about the noise to their teacher. That said, students might not use this strategy because they do not feel their teacher has any power to act on the noise.

Students who report being (affectively and physically) disturbed by noise report using coping strategies more frequently to avoid the noise. This result is similar to past findings suggesting that exposure to distressing noise resulted in the use of coping strategies [23]. Some variation did appear between noise types in relation to the use of coping strategies. Affective and bodily reactions to other children yelling and loud/intense sounds were more frequently related to coping behaviors than scraping/screeching sounds. This could be related to the nature of the noise; scraping/screeching sounds are often intermittent and short, and they could be perceived as less controllable and predictable. This could result in students taking less action to counter the noise. As stated earlier, perceived control over noise is a major factor of noise annoyance and of noise coping processes [38]. Future studies should include perceived control to explore that path further.

Differences were noted between boys and girls in this study. More girls reported being negatively affected by noise. This corroborates some previous findings suggesting girls are subjectively more affected by noise [2,3,51]; although, reference [13] failed to show any difference between boys and girls. Differences were also noted between primary and secondary school students in this study. Students in primary school reported more frequent noise exposure and more frequently experiencing bodily sensations to noise when compared to secondary school students. These results are similar to past findings suggesting younger students are more sensitive to noise [3,9,15]. However, Pirilä et al. [16] found older students to be more annoyed by noise, and Visentin and colleagues found younger students to perceive classroom noises as more pleasant than older ones, although their results might have been partly explained by an imbalanced data set [13]. Past results on affective and bodily reactions to noise are inconsistent and contradictory in regard to gender and age differences. That said, a plausible explanation for gender and age differences found in this study could be noise sensitivity. Noise sensitivity is a personal characteristic describing a person’s tolerance and reactivity to sound levels [52,53]. Noise-sensitive individuals are more reactive to noise and find noise more disturbing and psychologically more distressing [53,54]. Past studies, merely on adults, have linked noise sensitivity to gender and age [52,53]. It could be hypothesized that these factors also play a role in subjective reaction to noise in children populations. In this study, large inter-individual differences were observed in regard to affective and bodily reactions. These differences could be partly explained by noise sensitivity, gender, and age. That said, other factors may play a role, such as perceived control, the use of coping strategies, better recollection of negative feelings, and, most importantly, cognitive vulnerability [3,37,38]. Indeed, students with less developed inhibition skills (capacity to cognitively switch their attention to what is important) could be more affected by the noise [36]. Past research has shown that the perceived interference of noise with an ongoing task is related to negative appraisals of the noise [14,18,19,38]. Students who are aware of the noise interfering with what they are doing could have stronger negative reactions to that noise.

Participation in this study was voluntary for the classes and teachers. For this reason, unfortunately, some secondary school levels were not surveyed. The small number of schools in the sample, including only one secondary school, limits the generalization of the findings. It is unclear if the differences found in this study between primary school and secondary school could be attributed to the school itself or to the age of the students, as stated earlier. However, the most significant limitation of this study is the questionnaire. Waye et al. [45] created and validated their questionnaire for preschool-aged children. In their validation study, the questionnaire was administered as an interview by a qualified researcher, and the scales and figures of the questionnaire were used as visual support on show cards. In this study, children were older than in Waye and colleagues’ [45] samples, and the questionnaire was self-administered by the students. The students were judged old enough to read and understand the questions by themselves. To make sure the questions could be understood without adult help, a pilot study was conducted, and the questionnaire was tested with 20 school-aged children from primary and secondary levels. The pilot study concluded the questionnaire was correctly understood for students grade 4 and up. Data from the pilot study are not included in this article. That said, the Inventory of Noise and Children Health questionnaire is, to this date, not validated for the age group of this study. The lack of standardized and validated questionnaires for school-aged children on subjective reaction to noise in school settings is problematic and limits comparison between study results. Validated and standardized instruments on affective and bodily reactions to noise should be created to improve the generalization of study findings.

On average, students in this study have a neutral or negative affective reaction to noise, feel certain types of noise in their body, and sometimes use noise coping strategies but rarely experience noise-related symptoms such as headache, tummy ache, or a hoarse voice. The results of this study thus suggest that a nonnegligible proportion of students are subjectively disturbed by noise. We did, however, also find that a nonnegligible proportion of children reported positive feelings towards especially other children yelling, corroborating Visentin and colleagues’ findings that children report being pleasantly affected by hearing children’s voices from other classes [13]. This finding is interesting because past research on school quality and students’ learning has overlooked subjective perception in favor of acoustic and cognitive studies. It underlines the complex nature of noise perception and the need for studies also focusing on the positive aspects of soundscapes in schools [12] and the link between sound perception and the meanings individuals attach to the sounds [20]. Future studies should focus on the meanings, experiences, and perspectives of all children with qualitative research and guide the development of student-centered awareness-raising programs.

## 5. Conclusions

Few studies have been conducted on students’ subjective perception of noise in school environments. The present study has aimed to respond to that lack in the literature by exploring students’ insight into their affective and bodily reactions to three different types of sounds common in school environments (other children yelling, loud and intense sounds, and scraping and screeching sounds), as well as students’ coping behaviors and their experience of symptoms that could be related to noise exposure. Following Astolfi et al. [25], Dellve et al. [23], and Massonnie et al. [36], this study contributes to the slowly growing literature on the importance of including subjective perception in noise studies. Including said subjective perception helps better understand differences between individuals but also allows targeting individuals who would be at risk of impaired well-being in schools and who otherwise would be ignored. Awareness of affective and bodily reactions to noise should be implemented for teachers and students in school so they better understand their own reactions or the reactions of their peers to noise. Understanding subjective reactions to noise in learning settings is a step forward in an individual-centered way of dealing with noise.

## Figures and Tables

**Table 1:** Questions asked to the children by means of the INCH.

Question	Answer
How often do you hear $x^{a}$	5 point Likert scale ranging from Almost never to Always
When you hear $x^{a}$ , do you feel it inside you, or in your body?	Yes or No
Point to the child that looks like how you feel when you hear $x^{a}$ .	Two affective state scale
When there’s a lot of noise, what do you do?	
Do you go away?	5 point Likert scale ranging from Never to Always
Put your hands over your ears?	5 point Likert scale ranging from Never to Always
Tell the teacher?	5 point Likert scale ranging from Never to Always
Do you raise your voice to be heard?	5 point Likert scale ranging from Never to Always
How have you been feeling in school in the past few days?	
Have you had a headache?	5 point Likert scale ranging from Never to Always
Have you had a tummy ache	5 point Likert scale ranging from Never to Always
Have your voice been hoarse?	5 point Likert scale ranging from Never to Always

^*a*^ Questions asked for the three types of noise; children yelling, loud and intense noise, and scraping or screeching noise.

**Table 2:** Sample characteristics regarding school level and gender.

School Level	Girls	Boys	Others/Don’t Want to Tell	Missing Data	Total
Primary 4	26 (47.3%)	26 (47.3%)	3 (5.5%)		55 (13.5%)
Primary 5	51 (42.5%)	48 (40%)	5 (4.2%)	16 (13.3%)	120 (29.4%)
Primary 6	44 (42.3%)	56 (53.8%)	4 (3.8%)		104 (25.5%)
Secondary 2	51 (51%)	38 (38%)	11 (11%)		100 (24.5%)
Secondary 5	16 (55.2%)	12 (41.4%)	1 (3.4%)		29 (7.1%)
Total	188 (46.1%)	180 (44.1%)	24 (5.9%)	16 (3.9%)	408 (100%)

**Table 3:** The proportion of children experiencing bodily reactions to three types of sounds as a function of gender, school level, and body area.

Other Children Yelling				
	Head	Mid-Body	Lower Body	General
Girls (n = 188)	72 (38.3%)	31 (16.5%)	2 (1.1%)	85 (45.7%)
Boys (n = 180)	37 (20.6%)	25 (13.9%)	5 (2.8%)	57 (31.8%)
Primary (n = 275)	81 (29.5%)	39 (14.2%)	6 (2.2%)	112 (41%)
Secondary (n = 129)	37 (28.7%)	18 (14%)	1 (0.8%)	42 (32.8%)
Total (n = 404)	118 (29.2%)	57 (14.1%)	7 (1.7%)	154 (38.1%)
Loud and intense sounds				
	Head	Mid-body	Lower body	General
Girls (n = 188)	97 (51.6%)	45 (23.9%)	1 (0.5%)	122 (64.9%)
Boys (n = 177)	72 (40.7%)	33 (18.6%)	6 (3.4%)	96 (54.2%)
Primary (n = 275)	132 (48%)	67 (24.4%)	7 (2.6%)	180 (65.5%)
Secondary (n = 128)	52 (40.6%)	20 (15.6%)	1 (0.8%)	60 (46.9%)
Total (n = 403)	184 (45.7%)	87 (21.6%)	8 (2%)	240 (59.6%)
Scraping and screeching sounds				
	Head	Mid-body	Lower body	General
Girls (n = 185)	102 (55.1%)	65 (35.1%)	13 (7%)	131 (70.8%)
Boys (n = 178)	79 (44.4%)	51 (28.7%)	16 (9%)	107 (60.1%)
Primary (n = 260)	140 (53.8%)	86 (33.1%)	17 (6.5%)	185 (71.2%)
Secondary (n = 127)	56 (44.1%)	41 (32.3%)	14 (11%)	72 (56.7%)
Total (n = 387)	196 (50.6%)	127 (32.8%)	31 (8%)	257 (66.4%)

**Table 4:** Spearman’s rank correlations between coping strategy use and affective reactions towards sounds.

	Coping Strategy			
Other children yelling	Leaving	Hands on ears	Telling teacher	Raising voice
glad/safe–sad/afraid	0.218 **	0.239 **	0.109 *	NS
kind/friendly–angry/irritated	0.208 **	NS	0.129 *	
Loud and intense sounds	Leaving	Hands on ears	Telling teacher	Raising voice
glad/safe–sad/afraid	0.237 **	0.251 **	0.197 **	NS
kind/friendly–angry/irritated	0.200 **	0.286 **	0.231 **	0.113 *
Scraping and screeching sounds	Leaving	Hands on ears	Telling teacher	Raising voice
glad/safe–sad/afraid	0.157 **	0.185 **	0.160 **	NS
kind/friendly–angry/irritated	0.192 **	0.213 **	NS	NS

* p<0.05 ; ** p<0.005 ; NS: Non significant.

**Table 5:** Spearman’s rank correlations between general symptoms and affective reactions towards sounds.

	General Symptoms		
Other children yelling	Headache	Tummy ache	Hoarse voice
glad/safe–sad/afraid	0.203 **	0.130 *	NS
kind/friendly–angry/irritated	NS	0.237 **	NS
Loud and intense sounds	Headache	Tummy ache	Hoarse voice
glad/safe–sad/afraid	212 **	0.133 **	0.124 *
kind/friendly–angry/irritated	0.132 **	0.151 **	NS
Scraping and screeching sounds	Headache	Tummy ache	Hoarse voice
glad/safe–sad/afraid	0.178 **	0.166 **	0.224 **
kind/friendly–angry/irritated	NS	0.108 *	0.130 *

* p<0.05 ; ** p<0.005 ; NS: Non significant.

## Data Availability

Due to ethical restrictions, data supporting this study is not shared publicly. Data can be shared upon reasonable request to the corresponding author.

## Supplementary Materials

The following supporting information can be downloaded at www.mdpi.com/xxx/s1, Table S1: The proportion of children reporting affective reactions to three types of sounds as a function of gender; Table S2: The proportion of children reporting different levels of use of coping strategies (1: never–5: always), as a function of school level (primary vs secondary); Figure S1: The proportion of children reporting different levels of affective reactions to loud and intense noise as a function of having or not bodily reactions to these sounds; Figure S2: The proportion of children reporting different levels of affective reactions to screeching scratching noise as a function of having or not bodily reactions to these sounds
