# Supplementary material for: Student’s Self-Reported Experience of Soundscape: The Link between Noise, Psychological and Physical Well-Being
Source: Int J Environ Res Public Health. 2024 Jan 11;21(1):0. doi: 10.3390/ijerph21010084 (PMC11154246; doi:10.3390/ijerph21010084)
Supplement: Supplementary file 1 [file ijerph-21-00084-s001.zip › ijerph-2757436-supplementary.pdf]

Table S1: The proportion of children reporting affective reactions to three types of sounds as a function of gender

Other children yelling						
Levels: 1=glad/safe to 5=sad/afraid						
Girls/boys	1	2	3	4	5	
Girls (n=182)	2.2%*	9.9%	72%	14.8%*	1.1%	$Fisher'sexact : \rho = 0.043$
Boys (n=177)	6.8%*	14.1%	69.5%	9.6%*	0%	
Total (n=359)	4.5%	12%	70.8%	12.3%	0.6%	
Levels: 1=kind/friendly to 5=angry/irritated						
Girls/boys	1	2	3	4	5	
Girls (n=183)	5.5%	9.8%	57.4%	23.5%	3.8%	NS
Boys (n=175)	5.7%	17.1%	61.1%	13.1%	2.9%	
Total (n=358)	5.6%	13.4%	59.2%	18.4%	3.4%	
Loud and intense sounds						
Levels: 1=glad/safe to 5=sad/afraid						
Girls/boys	1	2	3	4	5	
Girls (n=177)	2.8%*	12.4%	56.5%	26%*	2.3%	Fisher's exact: $\rho = 0.001$
Boys (n=171)	9,9%*	15,8%	60,8%	12,3%*	1,2%	
Total (n=348)	6.3%	14.1%	58.6%	19.3%	1.7%	
Levels: 1=kind/friendly to 5=angry/irritated						
Girls/boys	1	2	3	4	5	
Girls (n=180)	2.2%	9.4%*	53.3%	28.3*%	6.7%	$\chi^2(4) = 10.646$, $\rho = .031$
Boys (n=172)	5.8%	17.4*%	50.6%	23.3*%	2.9%	
Total (n=352)	4%	13.4%	52%	25.9%	4.8%	
Scraping and screeching sounds						
Levels: 1=glad/safe to 5=sad/afraid						
Girls/boys	1	2	3	4	5	
Girls (n=183)	4.9%	12.6%	49.7%	30.1%	2.7%	NS
Boys (n=173)	8.7%	7.5%	56.1%	26.6%	1.2%	
Total (n=356)	6.7%	10.1%	52.8%	28.4%	2%	
Levels: 1=kind/friendly to 5=angry/irritated						
Girls/boys	1	2	3	4	5	
Girls (n=182)	3.3%	9.3%	42.9%	34.1%	10.4%	NS
Boys (n=170)	8.2%	7.6%	46.5%	30%	7.6%	
Total (n=352)	5.7%	8.5%	44.6%	32.1%	9.1%	

*Post-hoc tests are significant at the Bonferroni adjusted value of $\rho < .005$

.

Table S2: The proportion of children reporting different levels of use of coping strategies (1: never - 5: always), as a function of school level (primary vs secondary).

Leaving the noise						
Primary/secondary	1	2	3	4	5	
Primary (n=258)	15.4%*	14.2%	27.7%	22.9%*	19.8%	$\chi^2(4, N = 384) = 31.556$, $\rho < .001$
Secondary (n=126)	27.8%*	38.9%*	24.6%*	6.3%*	2.4%	
Telling the teacher there is noise						
Primary/secondary	1	2	3	4	5	
Primary (n=255)	25.2%*	23.9%*	14.9%*	6.7%	2.4%	Fisher's exact: $\rho = .002$
Secondary (n=126)	72.8%*	13.5%*	7.1%*	4%	1.6%	
Raising one's own voice						
Primary/secondary	1	2	3	4	5	
Primary (n=253)	15.4%	14.2%	27.7%	22.9%	3.6%	NS
Secondary (n=123)	8.1%	10.6%*	31.7%*	34.1%	15.4%	

*Post-hoc tests are significant at the Bonferroni adjusted value of $\rho < .005$

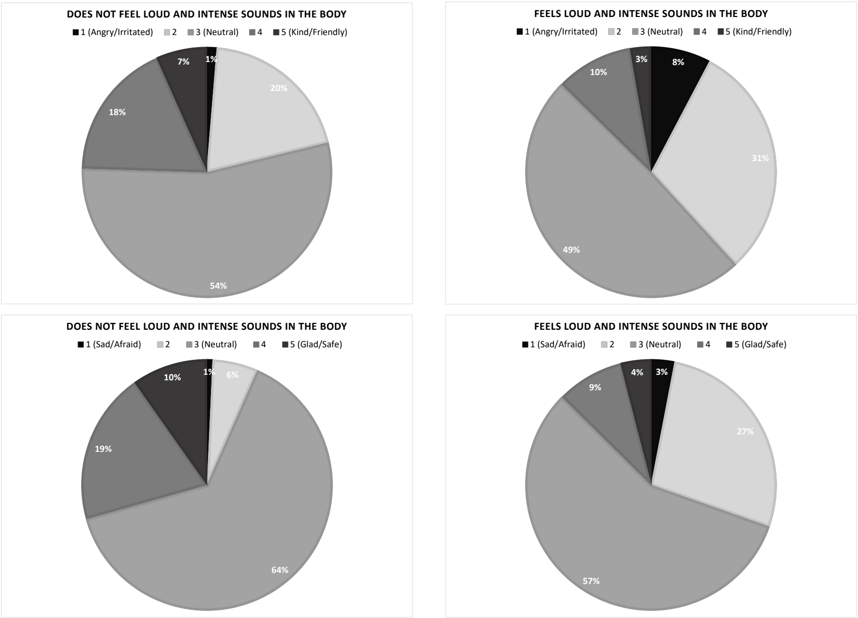

Figure S1: The proportion of children reporting different levels of affective reac-tions to loud and intense noise as a function of having or not bodily reactions to these sounds

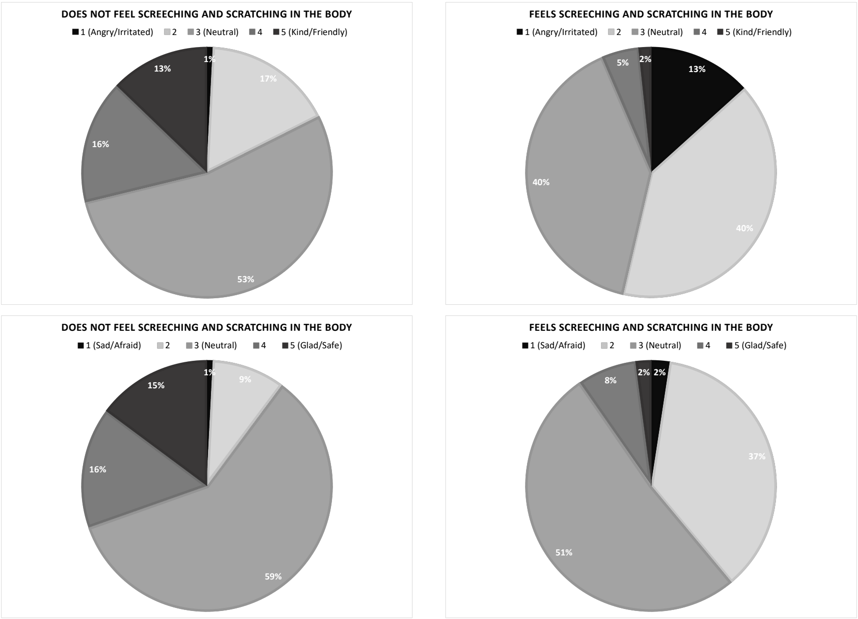

Figure S2: The proportion of children reporting different levels of affective re-actions to screeching scratching noise as a function of having or not bodily reactions to these sounds
